# The differential effects of sarcopenia and cachexia on overall survival for pancreatic ductal adenocarcinoma patients following pancreatectomy: A retrospective study based on a large population

**DOI:** 10.1002/cam4.5779

**Published:** 2023-03-20

**Authors:** Xiao‐ding Shen, Xing Wang, Zhen‐jiang Zheng, Yong‐hua Chen, Chun‐lu Tan, Xu‐bao Liu, Neng‐wen Ke

**Affiliations:** ^1^ Department of Pancreatic Surgery West China Hospital, Sichuan University Chengdu China

**Keywords:** cachexia, overall survival, pancreatectomy, pancreatic ductal adenocarcinoma, sarcopenia

## Abstract

**Objectives:**

Both cachexia and sarcopenia have been considered adverse predictors for prognosis in patients with pancreatic cancer; although sarcopenia and cachexia share some similarities, they are still defined as distinct nutritional conditions. We aimed to explore the differential impacts of sarcopenia and cachexia on prognosis for pancreatic ductal adenocarcinoma (PDAC) patients following radical excision.

**Methods:**

From January 2015 to May 2022, 614 patients undergoing surgery for PDAC were retrospectively included. Sarcopenia was defined as the L3 total skeletal muscle index below 52.4 cm^2^/m^2^ (men) and 38.5 cm^2^/m^2^ (women). Cachexia was classified according to the following criteria: involuntary weight loss >5% over the past 6 months, or weight loss >2% and BMI <20 kg/m^2^, or weight loss >2% and sarcopenia.

**Results:**

Of the 614 patients included in the analysis, 62% and 48% were diagnosed with sarcopenia and cachexia, respectively. Kaplan–Meier analysis showed that sarcopenia and/or cachexia were significantly associated with worse overall survival (OS) rather than worse recurrence‐free survival (RFS). Moreover, Cox regression analysis revealed that cachexia rather than sarcopenia was an adverse factor for OS in all PDAC patients. For poorly differentiated PDAC, both cachexia and sarcopenia were significantly associated with shorter OS. However, for moderately/well‐differentiated PADC, cachexia was an independent factor for adverse OS, but not sarcopenia.

**Conclusions:**

Sarcopenia and cachexia have different effects on OS for PDAC patients undergoing radical excision. This difference may provide some important information for preoperative management.

## Graphical abstract

We found that sarcopenia and cachexia have different effects on OS for PDAC patients undergoing radical excision by studying the data of 614 PDAC patients.

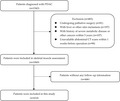

## 1 INTRODUCTION

Pancreatic ductal adenocarcinoma (PDAC) is a highly malignant tumor that is estimated to be the second leading cause of cancer‐related deaths by 2030.^1^ Although perioperative management for PDAC has improved in recent years, the prognosis only has minimally improved.^2^, ^3^ Radical excision remains the only curative therapy for PDAC^4^; however, the prognosis of PDAC after the operation is still poor. locoregional and/or distant recurrence develops in 80% of patients after curative resection and the 5‐year survival rate is less than 10%.^5^, ^6^ Multiple studies have proposed that tumor‐specific factors such as tumor status and histologic grade are associated with postoperative prognosis in patients with PDAC^7^, ^8^, ^9^; however, tumor‐specific factors are not decisive factors in the postoperative prognosis of patients with PDAC, and few studies have researched the association between postoperative prognosis of patients with PDAC and physical factors such as cachexia, muscle mass, and so on.

Cachexia is a complex syndrome characterized by progressive loss of weight, adipose, and skeletal muscle.^10^ PDAC is significantly associated with the development of cachexia and around 80% of patients with PDAC develop cachexia.^11^, ^12^, ^13^ Sarcopenia is defined as the physical component of syndromes which includes progressive loss of muscle mass and function^14^; indeed, most patients with PDAC would suffer from sarcopenia during the clinical course.^15^ Recently, some studies reported that cachexia or sarcopenia was an independent predictor of poor prognosis for many types of cancer, especially for PDAC.^16^, ^17^, ^18^, ^19^ Ya‐Chin Hou et al. found that cachexia and sarcopenia were poor predictors of overall survival (OS) for patients with advanced pancreatic cancer.^16^ Furthermore, some reports suggested that preoperative sarcopenia was associated with worse surgical outcomes in PDAC patients.^20^, ^21^ Sarcopenia shares some key points with cachexia such as weakness and systemic inflammation,^10^ but they are still defined as distinct nutritional conditions, some studies have revealed that sarcopenia and cachexia have different prognostic values for cancer patients.^16^, ^22^ However, no study has explored whether there are differential prognostic values of sarcopenia and cachexia for PDAC who receive radical excision.

Based on the above, we made a retrospective study to explore the impacts of sarcopenia and cachexia on postoperative prognosis in the same patients with PDAC. In this way, we could understand the differential prognostic values of sarcopenia and cachexia for PDAC patients who receive radical excision, improving the preoperative management for PDAC patients.

## 2 METHODS

### 2.1 Patient selection

Patients who underwent radical surgery for PDAC between January 2015 and May 2022 at the Department of Pancreatic Surgery of West China Hospital, Sichuan University were included. The eligibility criteria were as follows: (1) age ≥ 18; (2) radical surgery for PDAC; (3) available abdominal CT scans within 1 week before the operation. The exclusion criteria for this study were as follows: (1) patients undergoing palliative surgery; (2) with liver or other sites metastasis; (3) cases with a history of severe metabolic disease or other cancers within 5 years; (4) patients without any follow‐up information. Follow‐up was performed every 6 months and the last date of follow‐up was July 30, 2022.

### 2.2 Variables and definitions

The following clinical data were collected: patient age, gender, body mass index (BMI), comorbidities, stage of tumor, grade of tumor, length of hospital and intensive care unit (ICU) stay, postoperative complications, surgical procedure, hemoglobin (HGB), albumin (ALB), and adjuvant chemotherapy. Data on postoperative mortality and recurrence were also recorded. The stage of tumor was classified based on the American Joint Committee on Cancer staging system (AJCC) 8th edition and overall postoperative adverse events were evaluated based on the Clavien–Dindo classification system. The primary outcomes of this study were OS and recurrence‐free survival (RFS). OS was defined from the first day after surgery until death or the last follow‐up, RFS was defined from the first day after surgery until recurrence or death or the last follow‐up.

### 2.3 Cachexia and sarcopenia assessment

Cachexia was classified according to the following criteria: Involuntary weight loss >5% over the past 6 months, or weight loss >2% and BMI <20 kg/m^2^, or weight loss >2% and sarcopenia.^23^ For sarcopenia assessment, two trained persons analyzed preoperative CT images using open‐source semi‐automated software (BMI measurement tools, version 1.0).^24^ The total skeletal muscle area at the third lumbar vertebra (L3) level was measured from two consecutive axial CT minutes of silence and then averaged. The Hounsfield unit threshold for muscle was −29 and +150.^25^ Then, L3 total skeletal muscle area was normalized for height to obtain L3 total skeletal muscle index (L3SMI): L3 total skeletal muscle area (cm^2^)/height (m^2^). The cut‐offs for L3SMI were 52.4 cm^2^/m^2^ for men and 38.5 cm^2^/m^2^ for women.^26^, ^27^, ^28^

### 2.4 Statistical analysis

Continuous variables were reported as mean values with a standard deviation (SD) and categorical variables were presented as frequency statistics (n and %). Statistical testing was conducted by *t*‐test or Mann–Whitney *U*‐test for continuous variable and chi‐squared test or Fisher's exact test for categorical variables. Survival curves were plotted using the Kaplan–Meier method and compared using a log‐rank test. Prognostic factors of survival were identified by the Cox proportional hazard model, any factors with a *p* ≤ 0.10 in the univariate analysis were included in the multivariate analysis. All analyses were performed using SPSS 25. All *p* values were two‐sided and *p* < 0.05 were considered statistically significant.

## 3 RESULTS

**FIGURE 1:**
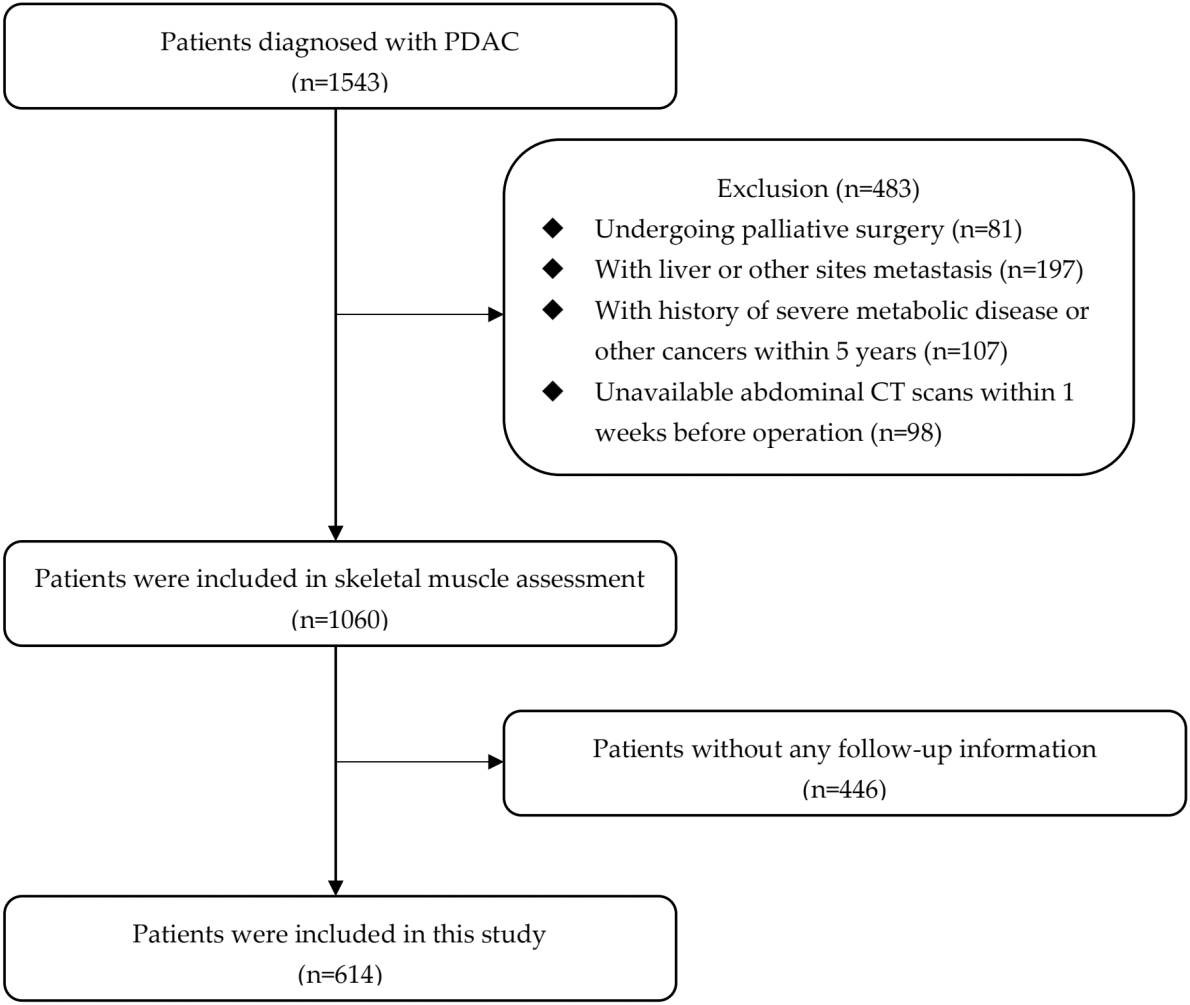
Flow diagram of patients.

From January 2015 to May 2022, a total of 1543 patients were potentially eligible and 929 patients were excluded who met the exclusion criteria and did not meet the inclusion criteria. Finally, 614 patients were available for this study (Figure 1). Baseline characteristics are presented in Table 1. The median OS time was 15 months.

**TABLE 1:** Baseline characteristics of patient groups according to sarcopenia and cachexia.

Variable^a^	Sarcopenia		*p*	Cachexia		*p*
	No	Yes		No	Yes	
Age (year)	59.10 ± 9.91	60.40 ± 10.52	0.13	60.17 ± 10.71	59.60 ± 9.84	0.49
Gender			**<0.01**			0.58
Female	140 (59.32%)	106 (28.04%)		132 (41.12%)	114 (38.91%)	
Male	96 (40.68%)	272 (71.96%)		189 (58.88%)	179 (61.09%)	
BMI (kg/m^2^)	23.65 ± 2.74	21.21 ± 2.61	**<0.01**	22.62 ± 2.88	21.63 ± 2.85	**<0.01**
Smoking			**<0.01**			0.73
No	174 (73.73%)	220 (58.20%)		208 (64.80%)	186 (63.48%)	
Yes	62 (26.27%)	158 (41.80%)		113 (35.20%)	107 (36.52%)	
Alcohol drinking			**<0.01**			0.45
No	180 (76.27%)	232 (61.38%)		211 (65.73%)	201 (68.60%)	
Yes	56 (23.73%)	146 (38.62%)		110 (34.27%)	92 (31.40%)	
Diabetes			0.25			0.60
No	199 (84.32%)	305 (80.69%)		266 (82.87%)	238 (81.23%)	
Yes	37 (15.68%)	73 (19.31%)		55 (17.13%)	55 (18.77%)	
Hypertension			0.33			0.49
No	180 (76.27%)	301 (79.63%)		255 (79.44%)	226 (77.13%)	
Yes	56 (23.73%)	77 (20.37%)		66 (20.56%)	67 (22.87%)	
Stage of tumor			0.09			0.53
0 + I	130 (55.08%)	182 (48.15%)		167 (51.71%)	145 (49.49%)	
II + III	106 (44.92%)	196 (51.85%)		154 (48.29%)	148 (50.51%)	
Grade of tumor			0.30			0.63
Poorly diff.	121 (51.27%)	210 (55.56%)		176 (54.83%)	155 (52.90%)	
Moderately/well diff.	115 (48.73%)	168 (44.44%)		145 (45.17%)	138 (47.10%)	
Postoperative chemotherapy^b^			**0.03**			0.36
No	77 (32.77%)	157 (41.53%)		117 (36.45%)	117 (40.07%)	
Yes	158 (67.23%)	221 (58.47%)		204 (63.55%)	175 (59.93%)	
Length of ICU stay (day)	1.53 ± 4.97	1.31 ± 2.57	0.71	1.40 ± 3.87	1.40 ± 3.47	0.32
Length of hospital stay (day)	16.59 ± 10.47	17.30 ± 11.79	0.73	16.09 ± 10.49	18.05 ± 12.06	**0.04**
Clavien–Dindo classification			0.34			0.08
0–II	208 (88.14%)	323 (85.45%)		285 (88.79%)	246 (83.96%)	
IIIA‐V	28 (11.86%)	55 (14.55%)		36 (11.21%)	47 (16.04%)	
Recurrence			0.98			0.85
No	124 (52.54%)	199 (52.65%)		170 (52.96%)	153 (52.22%)	
Yes	112 (47.46%)	179 (47.35%)		151 (47.04%)	140 (47.78%)	
Surgical procedure			0.31			**<0.01**
Pancreatoduodenectomy	155 (65.68%)	263 (69.58%)		202 (62.93%)	216 (73.72%)	
Total or distal pancreatectomy	81 (34.32%)	115 (30.42%)		119 (37.07%)	77 (26.28%)	
HGB (g/L)	126.08 ± 19.45	124.27 ± 18.68	0.25	127.28 ± 17.02	122.42 ± 20.66	**<0.01**
ALB (g/L)	41.72 ± 4.78	40.20 ± 5.07	**<0.01**	41.44 ± 4.79	40.05 ± 5.15	**<0.01**

*Note*: Bold values indicate that those factors are significantly associated with OS in the univariate or multivariate analysis.

Abbreviations: ALB, albumin; BMI, body mass index; diff, differentiated; HGB, hemoglobin; ICU, intensive care unit.

^a^ Continuous variables were reported as mean values with a standard deviation (SD) and categorical variables were presented as frequency statistics (n and %).

^b^ One missed the data.

According to the sex‐special cut‐offs for L3SMI, 378 patients were diagnosed with sarcopenia, accounting for 62% of all included patients. Sarcopenia was more prevalent among patients who smoked and drank, and males were more likely to suffer from sarcopenia. In addition, sarcopenia was significantly associated with lower BMI and lower ALB, and patients without sarcopenia before surgery were more likely to receive postoperative chemotherapy. In addition, 293 patients were diagnosed with cachexia accounting for 48% of all included patients. Patients with cachexia shared similar clinical characteristics to patients without cachexia, with the exception that they had significantly lower BMI, lower HGB, lower ALB, and longer hospital stay. The overall major adverse events rate (Clavien IIIA‐V) was similar in the sarcopenic group and non‐sarcopenic group as well as in the cachexic group and non‐cachexic group (11.86% vs. 15.55%, *p* = 0.34; 11.21% vs. 16.04%, *p* = 0.08, respectively).

**FIGURE 2:**
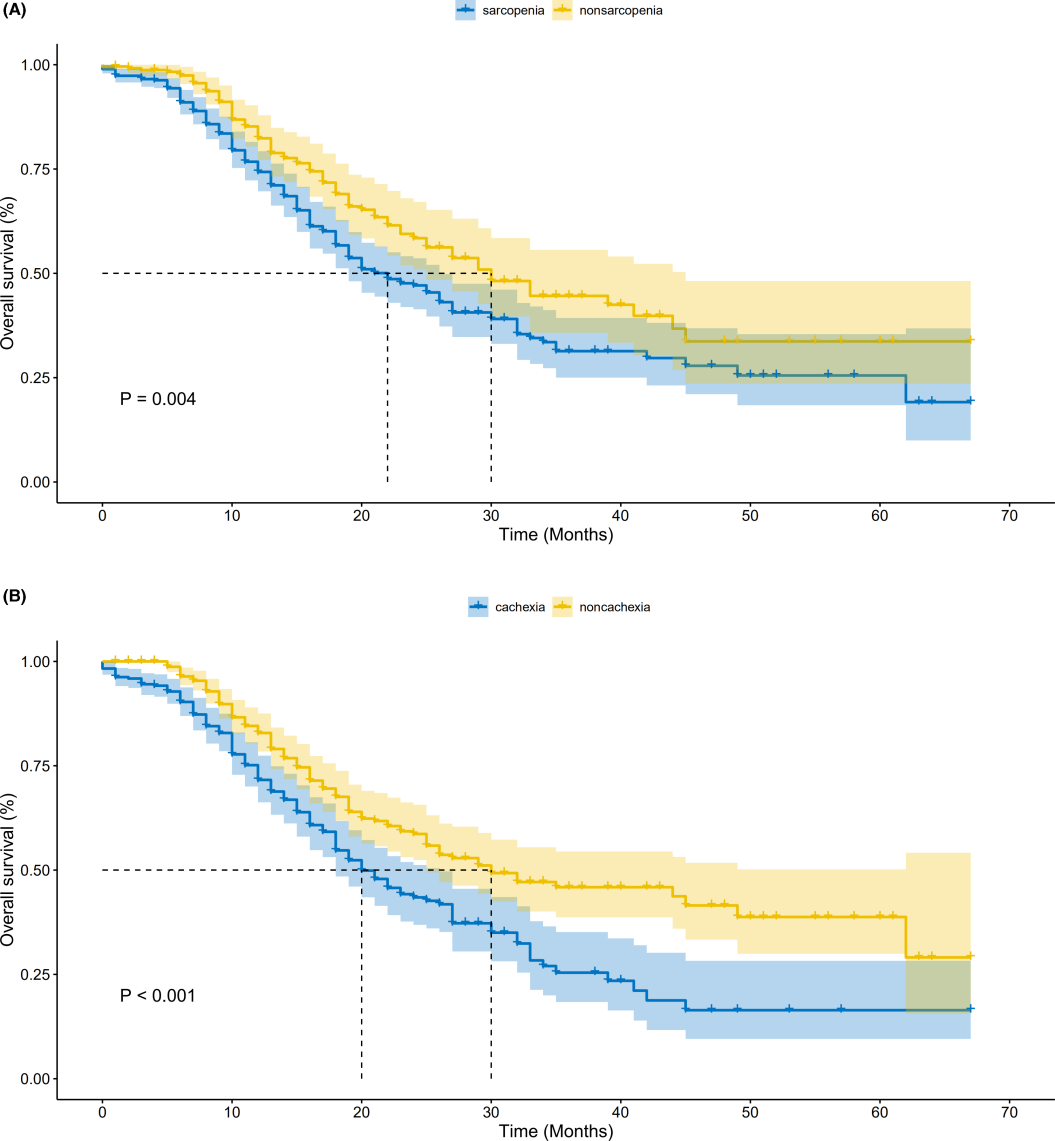
Kaplan–Meier curves for overall survival: (A) overall survival (OS) curves in patients with or without sarcopenia after radical excision for PDAC. (B) OS curves in patients with or without cachexia after radical excision for PDAC.

Kaplan–Meier analysis showed that both sarcopenia and cachexia were significantly associated with worse OS in patients with PDAC (log‐rank *p* < 0.05, Figure 2A,B). However, neither sarcopenia nor cachexia was associated with RFS in PDAC patients following radical excision (log‐rank *p* > 0.05, Figure S1A,B). Then, we explored whether there were differential effects of sarcopenia and cachexia on OS for PDAC patients following pancreatectomy; the univariate Cox regression analysis identified that both sarcopenia and cachexia were unfavorable factors for OS (Table 2). In addition, multivariate Cox regression analysis found that cachexia was significantly associated with worse OS in patients receiving radical excision for PDAC; however, the association between sarcopenia and OS did not reach significant (HR: 1.23, 95% CI: 0.93–1.63, *p* = 0.14, Table 2).

**TABLE 2:** Univariate and multivariate analysis of the invested factors for overall survival in all included patients.

Variable	Univariate analysis		Multivariate analysis	
	HR (95% CI)	*p*	HR (95% CI)	*p*
Age > 59, ref. ≤59^a^	1.05 (0.83–1.35)	0.67		
Gender, ref. female	1.20 (0.93–1.55)	0.15		
BMI > 22.22, ref. ≤22.22^a^	0.69 (0.54–0.89)	**<0.01**	0.88 (0.67–1.14)	0.32
Smoking, ref. no	1.14 (0.89–1.46)	0.31		
Alcohol drinking, ref. no	1.01 (0.78–1.31)	0.92		
Diabetes, ref. no	1.03 (0.75–1.41)	0.87		
Hypertension, ref. no	0.99 (0.73–1.33)	0.93		
Stage of tumor, ref. 0 + I	1.59 (1.24–2.04)	**<0.01**	1.66 (1.29–2.14)	**<0.01**
Grade of tumor, ref. poorly diff	0.53 (0.41–0.69)	**<0.01**	0.55 (0.42–0.71)	**<0.01**
Postoperative chemotherapy, ref. no	0.42 (0.33–0.54)	**<0.01**	0.38 (0.29–0.48)	**<0.01**
Clavien–Dindo classification, ref. 0‐II	1.19 (0.82–1.74)	0.37		
Recurrence, ref. no	1.63 (1.26–2.10)	**<0.01**	1.86 (1.43–2.42)	**<0.01**
Surgical procedure, ref. pancreatoduodenectomy	0.69 (0.51–0.91)	**0.01**	0.82 (0.61–1.10)	0.18
HGB > 126, ref. ≤126^a^	0.87 (0.68–1.12)	0.27		
ALB > 41, ref. ≤41^a^	0.85 (0.67–1.09)	0.20		
Sarcopenia, ref. no	1.46 (1.12–1.90)	**<0.01**	1.23 (0.93–1.62)	0.14
Cachexia, ref. no	1.64 (1.28–2.10)	**<0.01**	1.46 (1.14–1.89)	**<0.01**

*Note*: Bold values indicate that those factors are significantly associated with OS in the univariate or multivariate analysis.

Abbreviations: ALB, albumin; BMI, body mass index; CI, confidence intervals; diff, differentiated; HGB, hemoglobin; HR, hazard ratio; ref, reference.

^a^ The median was used as cut‐off value.

**FIGURE 3:**
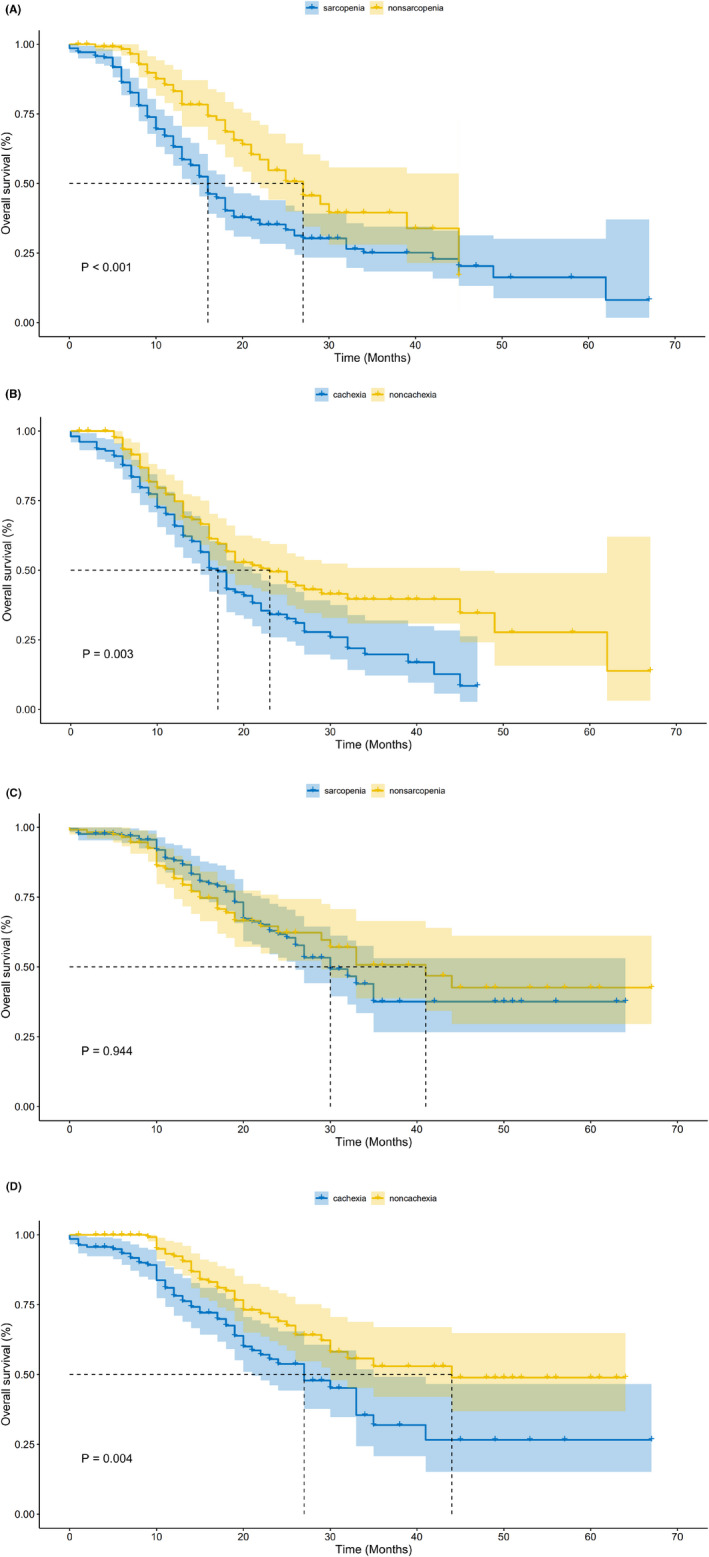
Kaplan–Meier curves for overall survival: (A) overall survival (OS) curves in patients with or without sarcopenia after radical excision for poorly differentiated PDAC. (B) OS curves in patients with or without cachexia after radical excision for poorly differentiated PDAC. (C) OS curves in patients with or without sarcopenia after radical excision for moderately and well‐differentiated PDAC. (D) OS curves in patients with or without cachexia after radical excision for moderately and well‐differentiated PDAC.

Additionally, we explored the impacts of sarcopenia and cachexia on survival in different grades of tumor groups. For the poorly differentiated group, Kaplan–Meier analysis presented that OS was significantly shorter in sarcopenia and cachexia patients, respectively (log‐rank *p* < 0.05, Figure 3A,B). Different results appeared in the moderately/well‐differentiated group (Figure 3C,D), cachexic patients had a shorter OS than non‐cachexic patients (log‐rank *p* < 0.01) while sarcopenic patients had comparable OS to non‐sarcopenic patients (log‐rank *p* = 0.94). Then, we further performed univariate and multivariate Cox regression analysis to identify the risk factors of OS in patients with different stages of PDAC. Cox regression analyses showed that cachexia was an independent risk factor of OS whether in the poorly differentiated group or moderately/well‐differentiated group. However, sarcopenia just was an independent risk factor of OS in the poorly differentiated group not in the moderately/well‐differentiated group (Tables S1 and S2).

## 4 DISCUSSION

Both cachexia and sarcopenia are negative factors for OS in cancer patients; previous studies focused on one or the other and identified that preoperative sarcopenia or cachexia was an independent factor of worse OS after surgery in patients with cancer.^29^, ^30^, ^31^, ^32^ Moreover, recent studies found that sarcopenia and cachexia had different clinical prognostic values. Cheng‐Le Zhuang et al. showed that sarcopenia had meaningfully prognostic value over cachexia for patients with early‐stage gastric cancer; however, the prognostic significance of cachexia exceeded sarcopenia for advanced gastric cancer.^22^ In addition, another article also found sarcopenia and cachexia had different impacts on the outcomes of advanced pancreatic cancer. Cachexia was an independent prognostic factor of worse OS for all patients with advanced pancreatic cancer and sarcopenia just was an unfavorable prognostic factor for OS in advanced pancreatic cancer within the high BMI group or the chemotherapy group.^16^ In this study, we also explored the differential impacts of sarcopenia and cachexia in the prognostic value after radical excision for PDAC; we found that cachexia rather than sarcopenia was associated with shorter OS in all patients with PDAC after radical excision. This was the first study to compare their prognostic values in the same PDAC patients following surgery based on a large single‐center population.

Although sarcopenia and cachexia share some similarities, they are still defined as distinct clinical conditions; sarcopenia is a syndrome characterized by progressive and generalized loss of muscle mass and function while cachexia is a complex syndrome that encompasses progressive weight loss and depletion of skeletal muscle mass and adipose tissue.^10^, ^14^, ^33^ Weight loss is a prerequisite forcachexia but not for sarcopenia, cachexia mainly presents both fat and fat‐free mass reduction but sarcopenia primarily presents muscle wasting.^34^, ^35^, ^36^ Furthermore, although systematic inflammation was observed both in sarcopenia and cachexia, cachexia usually is significantly associated with intense inflammatory processes while sarcopenia just often presents a slight or undetectable systematic inflammation.^10^, ^37^, ^38^, ^39^ Systematic inflammation is a negative prognostic factor of OS for patients with cancer.^40^, ^41^ Based on the above, we think cachexia represents a worse body condition than sarcopenia which may be the reason for the differential prognostic values of sarcopenia and cachexia.

Interestingly, we found that sarcopenia was an unfavorable prognostic factor for poorly differentiated PDAC rather than for moderately/well‐differentiated PDAC. This result was contrary to the previous findings. Both Moon Hyung Choi et al. and Yan‐Chih Peng et al. reported that preoperative sarcopenia identified on CT scan was strongly associated with shorter OS in all pancreatic cancer after surgery.^20^, ^31^ The differences among studies on the prognostic value of sarcopenia were probably due to the following factors: (1) the cut‐off value of sarcopenia was affected by different regions and ethnicities, and different cut‐off values of sarcopenia might lead to different diagnostic criteria; (2) different study cohorts might lead to different results, our study included a large population and just included patients with PDAC rather than other types of pancreatic cancer while other studies included all types of pancreatic cancer from a small population; (3) cachexia might be a potential confounder for the association between sarcopenia and OS, we performed Cox regression analysis containing both cachexia and sarcopenia in this study; however, other studies just brought sarcopenia into Cox regression analysis.

As we know, the degree of tumor malignancy is closely related to the grade of tumor, poorly differentiated PDAC was associated with a higher metastasis rate, faster tumor growth, and higher malignancy than moderately differentiated/well‐differentiated PDAC. We guess that cancer‐related sarcopenia is more severe in poorly differentiated PDAC than that in moderately differentiated/well‐differentiated PDAC, with the radical removal of the tumor, and the sarcopenia in patients with moderately differentiated/well‐differentiated PDAC can be largely reversed but not in patients with poorly differentiated PDAC. This might be the reason for the differential prognostic values of sarcopenia in the differential grade of PDAC.

Sarcopenia and cachexia represent the bad change in body composition, yet now no specific methods have been approved to treat these two conditions. Non‐pharmacological approaches and pharmacological approaches have been debated to treat sarcopenia and cachexia. For non‐pharmacological approaches, exercise and nutritional interventions are the most common methods. Exercise especially resistance exercise has the benefit of improving skeletal muscle strength and mass which helps improve the quality of life in patients with sarcopenia or cachexia.^12^, ^42^ The provision of adequate nutrition is a mainstay of treatment for cachexia, active nutrition interventions lead to a better quality of life in patients with cachexia.^43^ However, the effect of nutritional intervention for sarcopenia is still unclear; some studies present that dietary patterns such as adequate intake of protein and long‐chain polyunsaturated fatty acids help improve sarcopenia.^44^ However, what consists of an adequate intake of these key nutrients, and how to intake these nutrients in terms of timing and distribution throughout the day are still debated.^45^ For pharmacological approaches, the targeted drug has not been reported by any studies because of a poor understanding of the underlying mechanisms of cachexia and sarcopenia. Myostatin antibody and testosterone may have a beneficial effect on treating sarcopenia.^46^, ^47^ For cachexia, ghrelin receptor and melanocortin receptor 4 antagonists may have a positive therapeutic effect.^48^, ^49^, ^50^

There were several shortcomings in our study. First, this was a retrospective study in a single institute, a potential selection bias could exist. Second, the diagnosis of sarcopenia should include skeletal muscle, low muscle strength, and physical performance^51^; however, we could not assess the muscle strength and physical performance of included patients because this was retrospective research. This might have led to over‐ or underestimation of the prevalence of sarcopenia in our study. Finally, some patients might also receive neoadjuvant chemoradiotherapy, this factor might influence the results.

## 5 CONCLUSION

This study first demonstrated the different effects of sarcopenia and cachexia in the prognostic value within the same PDAC patients. Cachexia was an independent risk factor of OS after radical excision for patients with PDAC regardless of the grade of tumor, whereas sarcopenia just was a negative prognostic factor of OS after radical excision for PDAC patients within the poorly differentiated group not in the moderately differentiated/well‐differentiated group. This study makes us understand the difference between similar nutritional concepts more clearly and provides further information to stratify and intervene in preoperative patient risk in patients with PDAC.

## AUTHOR CONTRIBUTIONS

**Xiaoding Shen:** Conceptualization (equal); data curation (equal); formal analysis (equal); investigation (equal); methodology (equal); software (equal); writing – original draft (equal). **Xing Wang:** Conceptualization (equal); data curation (equal); formal analysis (equal); investigation (equal); methodology (equal); software (equal); writing – original draft (equal). **Zhenjiang Zheng:** Resources (equal). **Yonghua Chen:** Resources (equal). **Chunlu Tan:** Validation (equal); visualization (equal); writing – review and editing (equal). **Xu‐bao Liu:** Validation (equal); visualization (equal); writing – review and editing (equal). **Nengwen Ke:** Funding acquisition (equal); project administration (equal); supervision (equal).

## FUNDING INFORMATION

This study was supported by the National Natural Science Foundation of China (82002579).

## CONFLICT OF INTEREST STATEMENT

The authors declare that there is no conflict of interest.

## ETHICS STATEMENT

This study conformed to the provisions of the Declaration of Helsinki. All the patients included signed informed consent forms and agreed to data collection. It was approved by the ethics committee of West China Hospital, Sichuan University.

## Supporting information


Figure S1.

Table S1.

Table S2.

## Data Availability

All data generated or analyzed during this study are included in this published article.
