# Supplementary material for: The differential effects of sarcopenia and cachexia on overall survival for pancreatic ductal adenocarcinoma patients following pancreatectomy: A retrospective study based on a large population
Source: Cancer Med. 2023 Mar 20;12(9):10438–48. doi: 10.1002/cam4.5779 (PMC10225236; doi:10.1002/cam4.5779)
Supplement: Supplementary file 2 — Table S1. [file CAM4-12-10438-s003.docx]

**Supplementary table 1. Univariate and multivariate analysis of the invested factors for overall survival in patients with poorly differentiated PDAC**

| **Variable** | Univariate Analysis | | Multivariate Analysis | |
| --- | --- | --- | --- | --- |
|  | HR (95% CI) | *P* | HR (95% CI) | *P* |
| **Age >59, ref. ≤59 ^a^** | 1.21 (0.89-1.64) | 0.23 |  |  |
| **Gender, ref. female** | 1.00 (0.72-1.38) | 0.99 |  |  |
| **BMI >22.22, ref. ≤22.22^a^** | 0.66 (0.48-0.90) | **<0.01** | 0.84 (0.60-1.16) | 0.28 |
| **Smoking, ref. no** | 0.96 (0.70-1.32) | 0.81 |  |  |
| **Alcohol drinking, ref. no** | 0.86 (0.62-1.19) | 0.37 |  |  |
| **Diabetes, ref. no** | 0.97 (0.65-1.45) | 0.88 |  |  |
| **Hypertension, ref. no** | 0.97 (0.67-1.42) | 0.89 |  |  |
| **Stage of tumor, ref. 0+I** | 1.49 (1.09-2.04) | **0.01** | 1.45 (1.06-1.98) | **0.02** |
| **Postoperative chemotherapy, ref. no** | 0.41 (0.30-0.56) | **<0.01** | 0.43 (0.31-0.59) | **<0.01** |
| **Clavien Dindo Classification, ref. 0-II** | 0.87 (0.52-1.46) | 0.60 |  |  |
| **Recurrence, ref. no** | 1.12 (0.81-1.54) | 0.50 |  |  |
| **Surgical procedure, ref. pancreatoduodenectomy** | 0.74 (0.51-1.09) | 0.13 |  |  |
| **HGB >126, ref. ≤126 ^a^** | 0.88 (0.64-1.20) | 0.41 |  |  |
| **ALB >41, ref. ≤41 ^a^** | 0.86 (0.63-1.17) | 0.33 |  |  |
| **sarcopenia, ref. no** | 1.84 (1.30-2.61) | **<0.01** | 1.63 (1.13-2.34) | **<0.01** |
| **Cachexia, ref. no** | 1.58 (1.16-2.16) | **<0.01** | 1.39 (1.01-1.90) | **0.04** |

Abbreviations: BMI, body mass index; diff, differentiated; ref, reference; HR, hazard ratio; CI, confidence intervals; HGB, hemoglobin; ALB, albumin;

PDAC, Pancreatic ductal adenocarcinoma. ^a^The median was used as cut-off value.
