# Supplementary material for: The differential effects of sarcopenia and cachexia on overall survival for pancreatic ductal adenocarcinoma patients following pancreatectomy: A retrospective study based on a large population
Source: Cancer Med. 2023 Mar 20;12(9):10438–48. doi: 10.1002/cam4.5779 (PMC10225236; doi:10.1002/cam4.5779)
Supplement: Supplementary file 3 — Table S2. [file CAM4-12-10438-s002.docx]

**Supplementary table 2. Univariate and multivariate analysis of the invested factors for overall survival in patients with Moderately/well differentiated PDAC**

| **Variable** | Univariate Analysis | | Multivariate Analysis | |
| --- | --- | --- | --- | --- |
|  | HR (95% CI) | *P* | HR (95% CI) | *P* |
| **Age >59, ref. ≤59** | 0.91 (0.61-1.36) | 0.64 |  |  |
| **Gender, ref. female** | 1.29 (0.86-1.94) | 0.22 |  |  |
| **BMI >22.22, ref. ≤22.22^a^** | 0.76 (0.51-1.15) | 0.19 |  |  |
| **Smoking, ref. no** | 1.37 (0.91-2.07) | 0.13 |  |  |
| **Alcohol drinking, ref. no** | 1.19 (0.79-1.81) | 0.41 |  |  |
| **Diabetes, ref. no** | 1.06 (0.63-1.80) | 0.82 |  |  |
| **Hypertension, ref. no** | 1.05 (0.65-1.70) | 0.83 |  |  |
| **Stage of tumor, ref. 0+I** | 1.66 (1.11-2.50) | **0.02** | 1.62 (1.06-2.48) | **0.03** |
| **Postoperative chemotherapy, ref. no** | 0.41 (0.27-0.61) | **<0.01** | 0.39 (0.26-0.59) | **<0.01** |
| **Clavien Dindo Classification, ref. 0-II** | 1.90 (1.08-3.32) | **0.03** | 1.76 (0.99-3.14) | 0.05 |
| **Recurrence, ref. no** | 2.43 (1.59-3.73) | **<0.01** | 2.50 (1.63-3.84) | **<0.01** |
| **Surgical procedure, ref. pancreatoduodenectomy** | 0.73 (0.47-1.14) | 0.16 |  |  |
| **HGB >126, ref. ≤126 ^a^** | 0.85 (0.57-1.28) | 0.44 |  |  |
| **ALB >41, ref. ≤41 ^a^** | 0.94 (0.63-1.40) | 0.74 |  |  |
| **sarcopenia, ref. no** | 1.02 (0.67-1.53) | 0.95 |  |  |
| **Cachexia, ref. no** | 1.81 (1.20-2.72) | **<0.01** | 1.56 (1.04-2.36) | **0.03** |

Abbreviations: BMI, body mass index; diff, differentiated; ref, reference; HR, hazard ratio; CI, confidence intervals; HGB, hemoglobin; ALB, albumin; PDAC, Pancreatic ductal adenocarcinoma. ^a^The median was used as cut-off value.
